# Investigation of pathomechanisms of ventricular arrhythmias in cardiac laminopathies

**DOI:** 10.1186/1750-1172-10-S2-O7

**Published:** 2015-11-11

**Authors:** Antoine Muchir

**Affiliations:** 1 Sorbonne Universités, UPMC Univ Paris 06, INSERM UMRS974, CNRS FRE3617, Center for Research in Myology, Paris, France

## 

Mutations in *LMNA* are responsible for an aggressive form of dilated cardiomyopathy due to a high rate of malignant ventricular arrhythmias. Inter-cellular communication is essential for proper cardiac function. Mechanical and electrical activities must synchronize so that the work of individual cardiomyocytes transforms into the pumping function of the heart. This well-coordinated excitation-contraction coupling of the heart relies on an efficient inter-cellular communication, which is under the regulation of the intercalated discs. We focused on the understanding of the molecular mechanisms of components of intercalated disc re-localization in pathological context. For this, we investigated disease mechanisms and identify novel therapeutic targets, using an integrated series of models in cultured cells, mice and humans. Positive results will break new ground for future work towards developing novel treatment for malignant arrhythmias.

